# EHMTI-0187. A novel ATP1A2 mutation in a case of familial hemiplegic migraine with especially severe attacks

**DOI:** 10.1186/1129-2377-15-S1-B12

**Published:** 2014-09-18

**Authors:** E Martínez, R Moreno, L López-Mesonero, M Ruiz, I Vidriales, P Mulero, N Téllez, A Guerrero, JJ Tellería

**Affiliations:** 1 Neurologist, Hospital Clínico Universitario de Valladolid, Valladolid, Spain; 2 Clinical Analysis, Hospital Clínico Universitario de Valladolid, Valladolid, Spain; 3 Neurology, Hospital Clínico Universitario de Valladolid, Valladolid, Spain; 4 Genetics, Hospital Clínico Universitario de Valladolid, Valladolid, Spain

## Introduction

Familial hemiplegic migraine (FHM) is a rare disorder characterized by migraine attacks with motor weakness during aura phase. Mutations in CACNA1A, ATP1A2, SCN1A and PRRT2 genes have been described.

## Aim

To describe a novel mutation in ATP1A2 gene in a FHM case with especially severe and prolonged symptoms.

## Patient

22-year-old woman admitted due to migrainous headache and sudden onset right-side weakness and aphasia. Previous episodes during childhood. Her mother was diagnosed as hemiplegic migraine without genetic confirmation. Ten days before admission mild head trauma. On clinical exam, body temperature of 38ºC, diminished consciousness, left gaze preference, mixed aphasia, right facial palsy, right hemiplegia and left crural paresis. Urgent perfusion computed tomography showed hypoperfusion throughout all left cerebral hemisphere. No abnormalities in brain Magnetic Resonance and cerebrospinal fluid. In electroencephalogram diffuse slowing. Impaired consciousness and dysphasia began to improve three days after admission and mild dysphasia and right hemiparesis remained during 10 days. No recurrence during a six months follow-up.

## Results

Genomic DNA was extracted from peripheral blood and exons were amplified using PCR primers. Capture and massive sequencing of exons of candidate genes obtained using SureSelect Human All Exon 51 Mb kit (Agilent) and Hiseq2000 (Illumina) 30x tests. We identified an undescribed missense variant in heterozygous state in ATP1A2 gene (p.Thr364Met), pathogenic according to different prediction algorithms (SIFT, PolyPhen2, MutationTaster and Condel).

## Conclusion

Genotype-phenotype correlation in FHM caused by ATP1A2 mutations is not well defined. This new mutation might be linked to especially severe and long lasting attacks.

No conflict of interest.

